# Bone health up to five years after Roux-en-Y gastric bypass: a cross-sectional study during the COVID-19 pandemic

**DOI:** 10.1590/acb411426

**Published:** 2026-03-20

**Authors:** Alexandre Naegele de Oliveira, Taís Daiene Russo Hortencio, Daniela Vicinansa Monaco Ferreira, Karina Schiavoni Scandelai Cardoso dos Reis, Roberto José Negrão Nogueira

**Affiliations:** 1 Instituto de Pesquisa São Leopoldo Mandic – Faculdade São Leopoldo Mandic – Campinas (SP) – Brazil.; 2 Universidade Federal do Rio de Janeiro – Instituto de Ciências Médicas – Macaé (RJ) – Brazil.; 3 Universidade Estadual de Campinas – Faculdade de Ciências Médicas – Hospital de Clínicas – Campinas (SP) – Brazil.; 4 Universidade Estadual de Campinas– Faculdade de Ciências Médicas – Departamento de Clínica Médica – Campinas (SP) – Brazil.

**Keywords:** Gastric Bypass, Obesity, Bariatric Surgery, Bone Diseases, Metabolic, Osteoporosis, Pandemics

## Abstract

**Purpose:**

To evaluate bone health in obese patients undergoing surgery using the Roux-en-Y gastric bypass (RYGB) technique.

**Methods:**

In a sample of 22 obese patients undergoing RYGB surgery, evaluation of bone densitometry (BD) and plasma levels of calcium, phosphorus, magnesium, vitamin D (VITD), parathyroid hormone (PTH), alkaline phosphatase, and albumin was performed.

**Results:**

Signs of potential changes in calcification were detected in plasma tests, expressed as a decrease in VITD (46.4%) and an increase in PTH (50%). Three patients presented with bone framework damage. BD was unequivocally altered in three patients. A decrease in bone density was observed in 14 and 15 patients in the lumbar and femoral regions, respectively.

**Conclusion:**

Although PTH and VITD dosages did not show a direct correlation with BD, it was observed that approximately half of the patients had altered dosages of these hormones up to five years after surgery, and there was damage to the bone health expressed by BD. Loss to follow-up may have contributed to the increased risk of developing metabolic bone disease.

## Introduction

Obesity is a global public health concern. Since 1975, the number of obese people has tripled, with more than 650 million adults suffering from obesity in 2016^1^. Associated with continuous and effective multidisciplinary action, bariatric surgery results in immediate weight loss and maintenance of favorable long-term results^2,3^ as long as adequate monitoring occurs throughout life^4,5^.

The Roux-en-Y gastric bypass (RYGB) technique is used in a considerable number of total bariatric surgeries performed^2,3,6^ as it provides rapid weight loss and metabolic and hormonal improvements^7-10^. However, if the patient is not adequately monitored, malnutrition with different presentations and severities may occur. Changes in bone health are one of these manifestations, due to changes in the metabolism of calcium (Ca), phosphorus (P), vitamin D (VITD), and parathyroid hormone (PTH)^11^.

Recently, researchers evaluated changes in the Ca-VITD-PTH axis and bone mineral density (BMD) in long-term post-RYGB patients. The results demonstrated that most bariatric patients presented with disruption of the Ca-VITD-PTH axis, with secondary hyperparathyroidism being more frequent than hypovitaminosis D. Moreover, older patients and menopausal women had higher rates of low BMD^12^.

PTH is the main regulator of Ca homeostasis, stimulating the action of osteoclasts in the bone and controlling the release of Ca and P into the extracellular fluid^13^. Cellular Ca transport is greater in the duodenum and proximal jejunum and lower in the proximal colon, which explains the interference of RYGB in the absorption and transport of this mineral. VITD actively participates in the metabolism of Ca and P, which are fundamental to the maintenance of the bone framework. Although it is mostly obtained from sunlight, the final activation process requires hydroxylation in the liver and kidneys. Hypovitaminosis D is a common problem in the general population and is even more common in patients undergoing bariatric surgery, owing to dietary restrictions and reduced nutrient absorption^13-16^.

The COVID-19 pandemic has made access to health services difficult, hindering adequate monitoring and treatment of people with chronic diseases^17,18^. In this context, patients undergoing RYGB may have had difficulties with optimal follow-up. Considering the importance of monitoring bariatric patients, this study aimed to identify the impact of RYGB surgery on bone health.

## Methods

This cross-sectional prospective study was conducted on obese patients undergoing RYGB surgery between 2017 and 2019, under the care of a multidisciplinary team consisting of a surgeon, nutritionist, psychologist, and endocrinologist at a private clinic located in the city of Macaé, Rio de Janeiro, Brazil. All surgeries were performed by the same surgeon. All patients who underwent surgery during the study’s data collection period were included. Data collection continued until 2022, during the coronavirus disease (COVID-19) pandemic. Obese patients of both sexes who agreed to participate in the study by signing the informed consent form were included. Patients who were underage, had undergone surgical techniques other than RYGB, or who did not provide informed consent were excluded.

This study was approved by the Research Ethics Committee, under protocol number CAAE 55215522.2.0000.5374, and was conducted at the Instituto de Pesquisa São Leopoldo Mandic, in Campinas, São Paulo, Brazil.

The following data were collected from the analysis:

- Gneral identification: age, sex, current weight, and date of surgery;
- Anthropometric data, including height, weight on the date of surgery, weight, and change in body mass index (BMI) from surgery to data collection;
- Global health data: presence or absence of comorbidities, smoking, use of alcoholic beverages and medications, history of fractures, use of vitamin and mineral supplements (VITD and K, Ca, Mg, and P), duration of sun exposure, and frequency of physical activity.

The frequency of adequate exposure to sunlight was assessed following Michael Holick’s^19,20^ criteria, which considers aspects such as skin color, season, and latitude. As Brazil is a country with a mixed population, the present study considered sun exposure for more than 15 minutes between 8 a.m. and 11 a.m. or between 3 p.m. and 6 p.m. as suitable to produce VITD. The frequency of physical activity was assessed by applying the short version of the International Physical Activity Questionnaire (IPAQ)^21^, adapted for Brazil^22^, which classifies the individual in a ranking that varies from sedentary (less than 10 minutes of physical activity per week) to very active (minimum 30 minutes of vigorous activity five times a week).

Weight was measured using a BalMaK model 3 K 300F digital scale capable of measuring 2.5 to 300 kg, and height was measured using a horizontal stadiometer attached to the scale. Bone densitometry (BD) was performed using a DPX® Lunar Bone Densitometer (GE Medical Systems Lunar, United States of America) with two analyses: femoral neck (hip) and L1 to L4 vertebrae (lumbar).

The amount of VITD taken by the patients was compared with Guidelines on Vitamin D Replacement in Bariatric Surgery^23^. The results of plasma tests were collected:

- Ca: 8.3 to 10.3 mg/dL;
- PTH: 12 to 65 pg/mL;
- Alkaline phosphatase: 45 to 213 U/L;
- Albumin: 3.5 to 4.8 g/dL;
- VITD: healthy population under 60 years of age = greater than 20 ng/mL, population at risk or over 60 years of age = 30 to 60 ng/mL;
- P: 2.50 to 4.50 mg/L;
- Mg: 1.9 to 2.5 mg/dL^24,25^.

For statistical analysis, the data obtained were processed collectively and anonymously, systematized, and recorded in an electronic form on the Google Form platform, and subsequently tabulated and analyzed in the Microsoft Excel program. In the descriptive analyses, absolute frequencies, relative frequencies, minimum, maximum, mean, and standard deviations were used. The analyses were carried out using the R program with a significance level of 5%.

## Results

Among the 385 eligible patients, 22 agreed to participate in the study. Of these, 19 (86.4%) were women, had white skin color (59.1%) and was a mean age of 44 ± 10.2 years. The average loss of excess weight was 83 ± 22.5%. A total of 68.2% of patients had comorbidities, the most common being hypertension (31.8%) and hepatic steatosis (27.3%). Regarding habits, the participants were nonsmokers and 18.2% reported consuming alcoholic beverages. None of the patients had a history of fracture. In the BD carried out 49 ± 31 months after surgery, 86.4% of patients obtained a normal or adequate report, two (9.1%) had osteopenia and one (4.5%) had osteopenia associated with osteoporosis (Table 1).

**Table 1:** Descriptive analysis of the profile of study participants.

Variable	n (%)
**Sex**	
Female	19 (86.4)
**Skin color**	
White	13 (59.1)
Mixed race	6 (27.3)
Black	3 (13.6)
**Bone densitometry**	
Normal	19 (86.4)
Osteopenia	2 (9.1)
Osteopenia with osteoporosis	1 (4.5)
**Fracture history**	0 (0.0)
**Comorbidities**	15 (68.2)
**Type of comorbidities**	
Hypertension	7 (31.8)
Hepatic steatosis	6 (27.3)
Joint pain	1 (4.6)
Diabetes	1 (4.6)
Hemochromatosis	1 (4.6)
Hiatal hernia	1 (4.6)
Depression	1 (4.6)
Disc herniation	1 (4.6)
Osteoarthritis	1 (4.6)
**Habits**	
Smoking	0 (0.0)
Use of alcoholic beverages	4 (18.2)
**Variable**	Mean (standard deviation; minimum; maximum)
Age (years old)	44.0 (10.2; 26.0; 68.0)
Time between surgery and densitometry (months)	49.0 (13.3; 31.75; 56.75)
Time between surgery and exams (months)	50.00 (12.99; 34.5; 57.75)
Weight at the time of surgery	113.9 (20.0; 87.0; 166.2)
Excess weight (Kg)	47.9 (14.6; 30.2; 95.7)
Pre-operative body mass index (Kg/m2)	42.0 (4.4; 36.7; 51.9)
Current body mass index (Kg/m2)	28.1 (4.2; 22.4; 39.6)
Weight lost (Kg)	37.7 (11.1; 22.6; 76.2)
Excess weight loss (%)	83.0 (22.5; 42.7; 119.1)

Source: Elaborated by the authors.

A total of 63.6% of patients were taking vitamin and/or mineral supplementation (isolated or multivitamins). However, only 22.7% took adequate VITD supplementation, and only one patient (4.6%) received adequate Ca supplementation. Regarding the frequency of exposure to the sun, 86.4% exposed themselves to the sun, and of these, 22.7% had adequate exposure. Although all participants stated that they performed some physical activity, only 45.4% performed the recommended amount of it (Table 2).

**Table 2:** Descriptive analysis of the use and adequacy of vitamin and mineral supplements, adequacy of sun exposure, and physical activity practice of study participants.

Vitamin/mineral	Recommended need	Use	Use in the recommended amount
		n (%)	
Vitamin D	800 to 1,000 UI/day	14 (63.6)	5 (22.7)
Vitamin K	90 a 120 µg	8 (36.4)	0 (0.0)
Calcium	1,500 to 2,000 mg/day	10 (45.4)	1 (4.6)
Magnesium	320 to 420 mg/day	8 (36.4)	0 (0.0)
Phosphorus	700 mg/day	2 (9.1)	0 (0.0)
Supplement*	-	14 (63.6)	6 (27.3)
Sun exposure**	Skin-tone dependent	19 (86.4)	5 (22.7)
Physical exercise***	Intensity dependent	22 (100.0)	10 (45.4)

* Proper use of at least one supplement;

** more than 15 min/day, depending on the skin tone;

*** 150 to 300 min/week of moderate activity or 70 to 150 min/week of intense activity.

Source: Elaborated by the authors.

Table 3 presents the adequacy and inadequacy of the examinations performed by the patients. It was observed that 50% of patients had elevated PTH and 45% had VITD deficiency. Furthermore, 22.7% had inadequate magnesium levels. Conversely, more than 90% of patients had adequate results for other tests (Ca, P, albumin, and alkaline phosphatase).

**Table 3:** Descriptive analysis of the adequacy and inadequacy of calcium, phosphorus, parathyroid hormone, albumin, vitamin D, and magnesium tests.

Exam	Reference values	Adequate n (%)	Inadequate n (%)	Altered values	Frequency n (%)
Calcium	8.3 to 10.3 mg/dL	20 (90.9)	2 (9.1)	8.2	1 (4.5)
				10.4	1 (4.5)
Phosphorus	2.50 to 4.50 mg/dL	20 (90.9)	2 (9.1)	4.7	1 (4.5)
				4.9	1 (4.5)
Alkaline phosphatase	45 to 213 U/L	22 (100)	-	-	-
Parathyroid hormone	12 to 65 pg/mL	11 (50.0)	11 (50.0)	66	1 (4.5)
				68	1 (4.5)
				73	1 (4.5)
				76	1 (4.5)
				80	1 (4.5)
				80.3	1 (4.5)
				90	1 (4.5)
				107	1 (4.5)
				115	1 (4.5)
				131	1 (4.5)
				159.5	1 (4.5)
Albumin	3.5 to 4.8 g/dL	20 (90.9)	2 (9.1)	4.9	2 (9.1)
Vitamin D	30 to 60 ng/mL	12 (54.6)	10 (45.4)	16.7	1 (4.5)
				17	1 (4.5)
				19.2	1 (4.5)
				24	2 (9.1)
				24.1	1 (4.5)
				24.4	1 (4.5)
				26	1 (4.5)
				27.3	1 (4.5)
				28.9	1 (4.5)
Magnesium	1.9 to 2.5 mg/dL	17 (77.3)	5 (22.7)	1.5	1 (4.5)
				1.7	2 (9.1)
				1.6	1 (4.5)
				1.63	1 (4.5)

Source: Elaborated by the authors.

**Figure 1:**
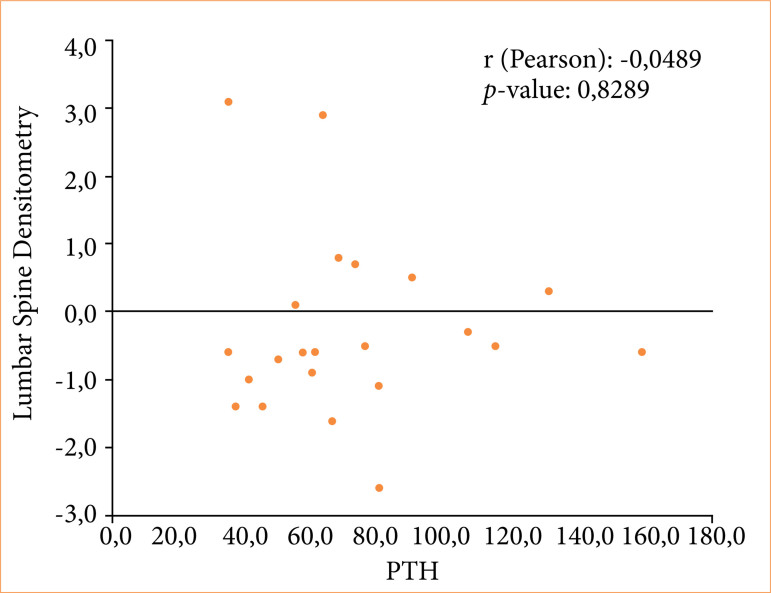
Correlation between lumbar spine densitometry and PTH of patients undergoing bariatric surgery using the Roux-en-Y gastric bypass technique.

**Figure 2:**
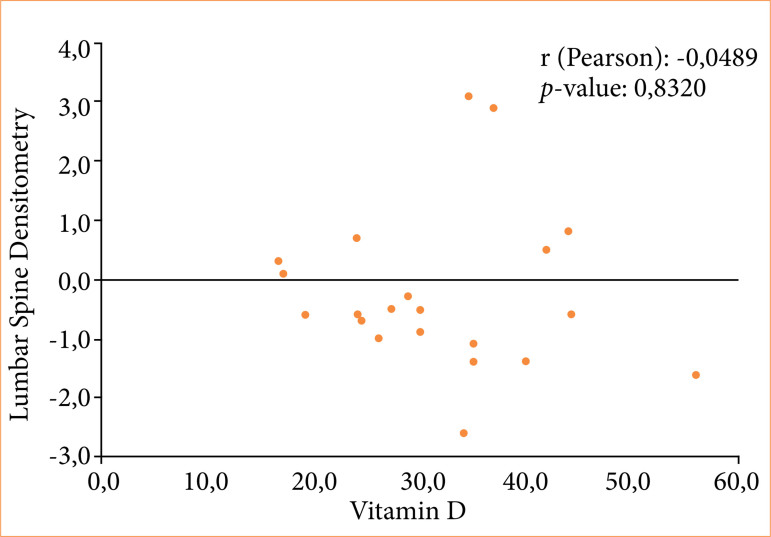
Correlation between lumbar spine densitometry and vitamin D of patients undergoing bariatric surgery using the Roux-en-Y gastric bypass technique.

**Figure 3:**
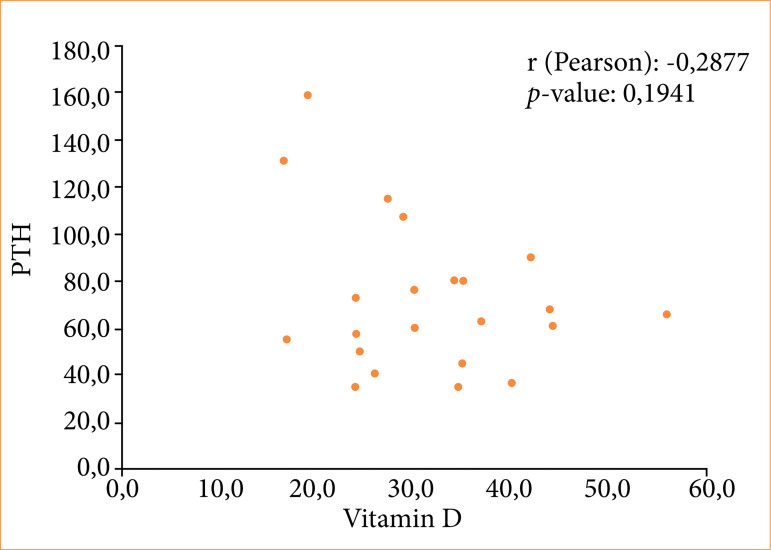
Correlation between PTH and vitamin D in patients undergoing bariatric surgery with the Roux-en-Y gastric bypass technique.

Weak correlations between lumbar spine densitometry and PTH (Pearson correlation coefficient r = -0.0489; *p* = 0.8289) (Fig. 1), lumbar spine densitometry and VITD (r = -0.0480; *p* = 0.8320) (Fig. 2), and PTH and VITD (r = -0.2877; *p* = 0.1941) (Fig. 3) were observed.

PTH: parathyroid hormone. Source: Elaborated by the authors.

PTH: parathyroid hormone. Source: Elaborated by the authors.

PTH: parathyroid hormone. Source: Elaborated by the authors.

## Discussion

Considering the particularities of follow-up in bariatric patients^5^ and the difficulties imposed by the SARS-CoV-2 pandemic^17^, the follow-up of bariatric patients during this period was increasingly compromised. However, the use of BD in the present study is relevant, as it is the gold standard for assessing bone health. Another notable aspect was that the entire cohort underwent surgery by a single surgeon using the same technique. All patients were monitored by the same multidisciplinary team.

Plasma mineral assessments were normal, but hormonal changes occurred in approximately half of the patients, elevated PTH in 50%, and decreased VITD in 46.4% of patients. Analysis of the three basic elements for preservation of the bone framework (physical activity, sun exposure, and VITD replacement) revealed that the three elements were insufficient. The inverse relationship between decreased VITD and elevated PTH is well known and occurs because of secondary hyperparathyroidism caused by VITD^15^ deficiency.

A study in pre- and post-menopausal women after RYGB demonstrated that chronic VITD deficiency causes secondary hyperparathyroidism and, consequently, bone loss^26^. In the present study, composed of 86.4% of women, it was observed that all individuals who had lower levels of VITD had high PTH and adequate Ca levels.

Serum PTH concentration is inversely associated with BD^27^. When analyzing the bone health of our patients using the BD of the lumbar spine, which demonstrates bone loss earlier than the femur, it is notable that the presence of osteopenia/osteoporosis occurred in 13.6% of patients. However, when considering the hormones involved in bone health, PTH and VITD, evidence of secondary hyperparathyroidism was observed in approximately 50% of the participants.

A previous study aimed to elucidate the role of existing serum biomarkers of VITD metabolism in assessing the risk and prevention of fractures in relatively healthy multi-ethnic populations and demonstrated a direct relationship between serum levels of VITD and BD and a negative correlation between PTH and VITD when the latter was lower than 30 ng/mL. In the same study, serum PTH levels showed a significant negative association with BD in the hip and lumbar spine^27^.

Only 22.7% of patients studied used adequate VITD supplementation, and further studies suggest that the need for optimal VITD supplementation is greater after bariatric surgery than in other non-surgical populations^28,29^.

In the present study, although a weak correlation was demonstrated between the BD of the lumbar spine and PTH and VITD, by visual analysis of Figs. 1–3, it was possible to verify that both high PTH and low VITD were present when the BD was lower. This allowed us to speculate that it would only be a matter of time before BD became unequivocally altered in our cohort, should low levels of VITD and inadequate VITD replacement occur. In fact, a lack of visible bone loss on BD does not mean that it will not occur, and it is essential not to allow negative bone deposits to persist over time. This is more relevant when observing that the average age of women was 44 ± 10.2 years old, as levels may decrease further over time in the presence of menopause^26^ if Ca and VITD supplementation dosages are not adjusted based on the life cycle needs. A longitudinal cohort study that evaluated the relationship between VITD status and PTH over five years after gastric bypass concluded that each 1-nmol/L increase in serum 25-hydroxyvitamin D level was related to a decrease in PTH level of 0.031 pmol/L during follow-up and viceversa^30^.

Between 2020 and 2022, the SARS-CoV-2 pandemic made it difficult to follow up on patients with chronic diseases^31,32^. Aspects such as the justifiable great worry of patients seeking medical services for fear of contamination and factors inherent to the health services that made their efforts to combat the pandemic explain, in part, the lack of follow-up of all patients with chronic diseases, as reflected in this study.

Initially, 385 patients were eligible to participate in the study, based on the total number of individuals who underwent bariatric surgery at the institution during the study period. However, due to the worsening of the COVID-19 pandemic, there was a significant loss to follow-up, with many patients failing to return for postoperative assessments, which compromised adherence to the study. Thus, although an adequate sample size was initially conceived to ensure sufficient statistical power, the extraordinary circumstances of the pandemic affected effective data collection and the final participation rate.

Regarding the limitations of this study, it involved a cross-sectional cohort with a small sample size, which made it impossible to infer cause-and-effect relationships in association analyses. However, we were able to observe a lack of adherence in several aspects that are essential for the patient’s bone health, which, in the medium and long term, will result in severe bone demineralization with consequent morbidity and worsening of the quality of life if no measures are taken. However, it is important to highlight that in this study, the best tool for diagnosing bone condition was used and that the same surgical technique was used by the same surgeon for all participants involved; therefore, there was no heterogeneity in the technique and/or follow-up. A more robust statistical analysis could have been performed if a greater number of cases had been included. This was impossible due to the pandemic.

## Conclusion

Patients who underwent RYGB followed by treatment during the COVID-19 pandemic showed signs of bone damage. Loss to follow-up may have contributed to the development of metabolic bone disease. Although PTH and VITD levels did not show a direct correlation with BD, hormonal changes highlight the importance of strict multidisciplinary follow-up during the postoperative period.

## Data Availability

All data sets were generated or analyzed in the current study.
